# Supplementary material for: Automated multiplex genome-scale engineering in yeast
Source: Nat Commun. 2017 May 4;8:15187. doi: 10.1038/ncomms15187 (PMC5418614; doi:10.1038/ncomms15187)
Supplement: Supplementary Information — Supplementary Figures 1-10, supplementary Tables 1-5 and supplementary References. [file ncomms15187-s1.pdf]

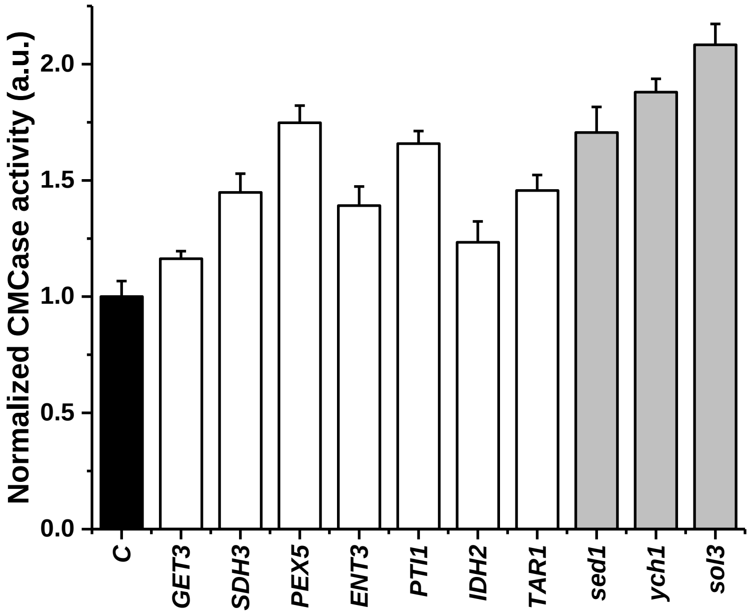

Supplementary Figure 1 | Genetic mutations conferring improved EGII-display levels estimated using hydrolysis activities of carboxymethyl cellulose (CMC) substrates. Overexpression and knockdown targets are listed as white and grey bars, respectively. *C* denotes the control strain (CAD-EGII) harboring an empty pRS416 plasmid. Error bars represent the mean ± s.d. from biological replicates (n=3).

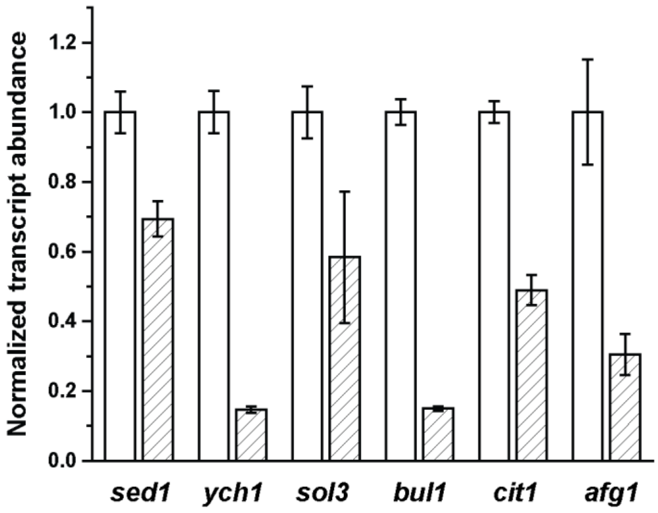

Supplementary Figure 2 | Estimation of gene silencing efficiency in yeast mutants. Genetic knockdown was mediated by plasmids containing expression cassettes of full-length antisense mRNAs on a pRS416 backbone. Transcript abundance of the knockdown mutants (patterned bars) was normalized to the control strains (no pattern bars) containing an empty pRS416 plasmid. The *ACT1* gene was used as the internal control for RT-qPCR experiments. Averages are defined as the central values, and error bars represent the mean ± s.d. from biological replicates (n=3, and technical triplicates were included for each sample).

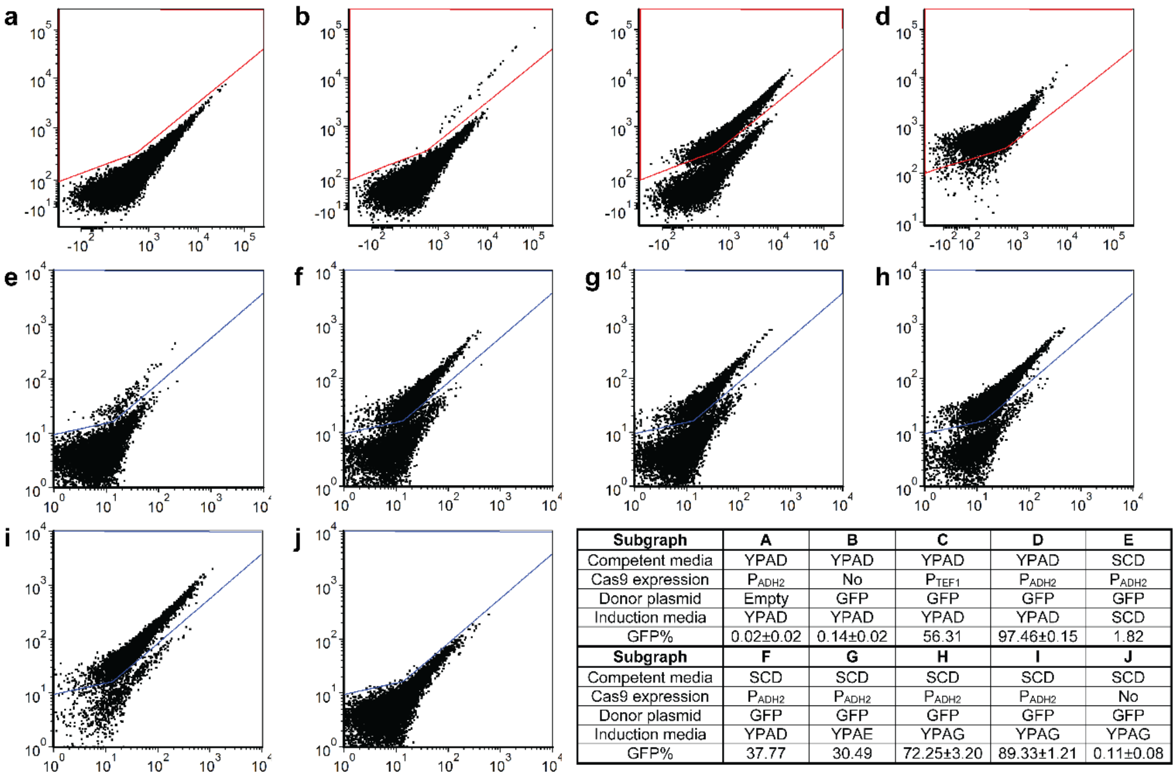

Subgraph	A	B	C	D	E
Competent media	YPAD	YPAD	YPAD	YPAD	SCD
Cas9 expression	P_{ADH2}	No	P_{TEF1}	P_{ADH2}	P_{ADH2}
Donor plasmid	Empty	GFP	GFP	GFP	GFP
Induction media	YPAD	YPAD	YPAD	YPAD	SCD
GFP%	0.02±0.02	0.14±0.02	56.31	97.46±0.15	1.82
**Subgraph**	**F**	**G**	**H**	**I**	**J**
Competent media	SCD	SCD	SCD	SCD	SCD
Cas9 expression	P_{ADH2}	P_{ADH2}	P_{ADH2}	P_{ADH2}	No
Donor plasmid	GFP	GFP	GFP	GFP	GFP
Induction media	YPAD	YPAE	YPAG	YPAG	YPAG
GFP%	37.77	30.49	72.25±3.20	89.33±1.21	0.11±0.08

Supplementary Figure 3 | CRISPR-assisted δ integration of a GFP reporter. Representative flow cytometry plots are provided for quantification of GFP-positive populations by depicted gates after donor plasmid curation. The *y*-axis denotes GFP fluorescence, while the *x*-axis denotes yeast cell autofluorescence measured using a phycoerythrin (PE) channel. (**a-d**) In earlier designs, YPAD was used to prepare competent cells. Only few transformants (<10^2 CFUs per µg plasmid DNA) can be obtained after plasmid transformation, probably due to the fact that P_{ADH2} is not strictly repressed by glucose in rich medium. (**e-j**) Higher transformation efficiency (~10^4 CFUs per µg plasmid DNA) was achieved using synthetic medium with glucose (SCD) for competent cell preparation, although integration efficiency was reduced by about three-fold (**d** and **f**). (**a**) Negative control with the CAD-P_{ADH2}-Cas9 strain and an empty donor plasmid. (**b)** Small GFP positive population with the CAD strain (without Cas9 expression) and the GFP donor plasmid. GFP fluorescence may be resulted from δ integration or incomplete plasmid curation. (**c**) and (**d**) High percentiles of GFP positive population enabled with Cas9 expression and the GFP donor. Higher integration efficiency was achieved when Cas9 was expressed from P_{ADH2} compared with P_{TEF1}, and hence the CAD-P_{ADH2}-Cas9 strain was used for later studies. (**e**)-(**h**) Optimization of induction media. Different media types (synthetic medium and rich media) and carbon sources (D: 2% glucose, E: 2% ethanol, G: 2% galactose) were examined. YPAG was chosen as the best induction medium. (**i**) The second round of integration with the GFP donor plasmid using cells from (**h**) as parent. (**j**) Two rounds of integration with the GFP donor plasmid in the CAD strain (without Cas9 expression). A table was provided to summarize experiment conditions and statistics from (**a-j**). Error bars represent the mean ± s.d. from biological replicates (n=3). Average values without error bars are calculated from biological replicates (n=2). Two independent experiments were performed for all panels.

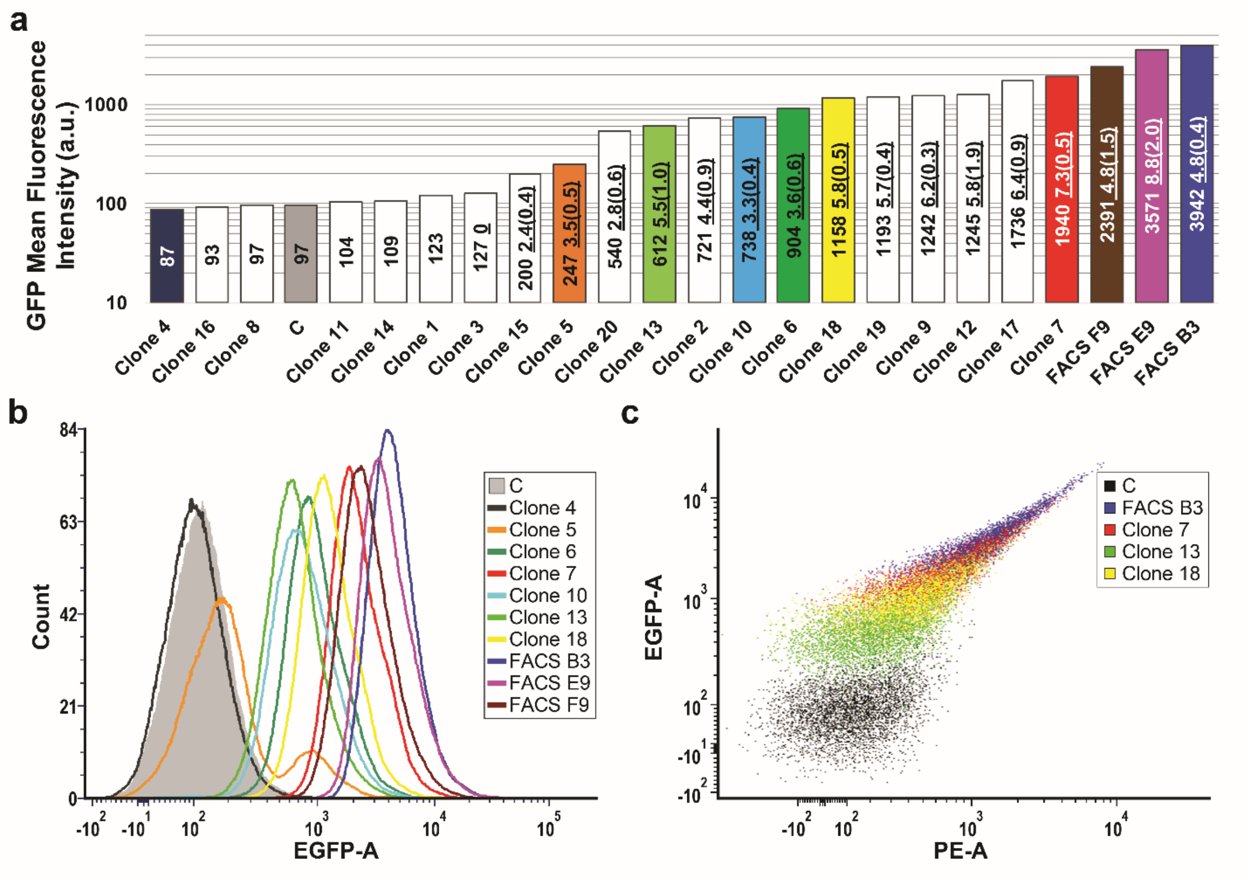

Supplementary Figure 4 | Characterization of individual clones after CRISPR-assisted δ integration of a GFP reporter. From the cell population after the first round of integration (**Round 1** in **Fig. 3c** and **Supplementary Fig. 3h**), individual clones were isolated either randomly after streaking on an agar plate (denoted as Clone #) or using FACS (denoted as FACS #). Twenty randomly picked clones and the top 3 brightest clones from FACS are presented. For brevity and clarity, only selected clones from panel (**a**) are included in (**b**) and (**c**), and the same color code is used in (**a**)-(**c**). (**a**) Mean GFP fluorescence intensities by flow cytometry. Clones with GFP fluorescence higher than Clone 3 were considered GFP-positive based on histogram and qPCR results. Mean GFP intensity values are in the normal font, and integration copy numbers quantified using qPCR are underlined with the format of mean(s.e.m) (**b**) Histogram results of flow cytometry. (**c**) Dot plots of flow cytometry. Two independent flow-cytometry experiments were performed with similar trends observed. For qPCR, technical replicates (n=3) of two template concentrations (3 ng μL^{-1} and 0.3 µg uL^{-1}) were performed using the *ALG9* gene as reference.

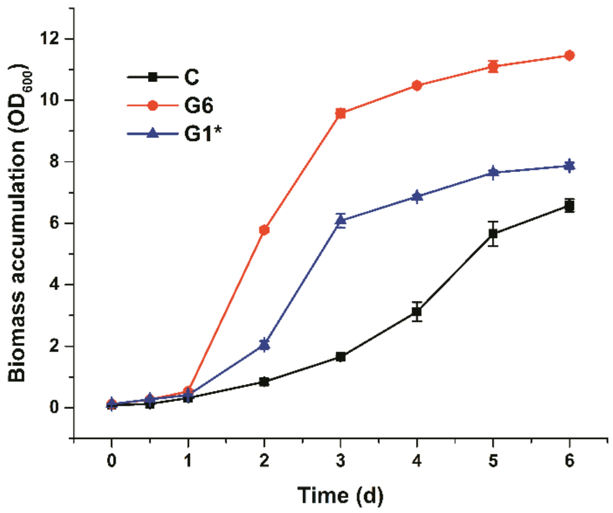

Supplementary Figure 5 | Improved glycerol utilization by yeast mutants. Error bars represent the mean ± s.d. from biological replicates (n=3). Averages are plotted as center values. Two independent experiments were performed with the same trend observed.

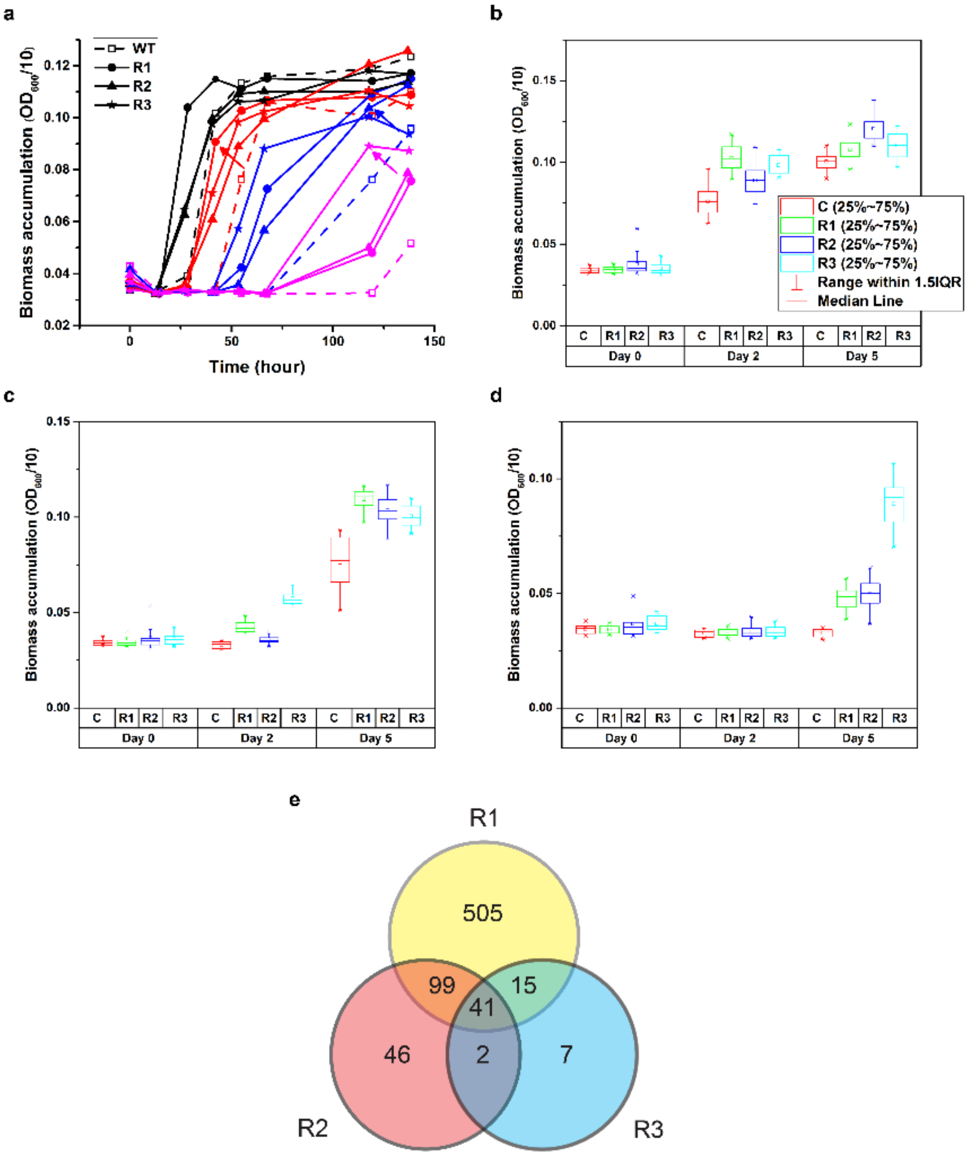

Supplementary Figure 6 | Screening of yeast mutants with improved acetic acid (HAc) tolerance. (**a**) The yeast libraries (solid line) and the control strain (dashed line) were cultivated aerobically in synthetic media supplemented with 0.8% (black), 0.9% (Red), 1.0% (blue) and 1.1% (magenta) HAc. Selection of cell cultures from the first (R1, red circle, 0.9%), second (R2, blue triangle, 1.0%) and third (R3, magenta star, 1.1%) rounds was indicated with arrows. Mean values from technical replicates (n=16) are plotted. (**b-d**) Comparison of biomass accumulation of cell populations from different rounds in the presence of 0.9% (**b**), 1.0% (**c**) and 1.1% (**d**) (v/v) HAc. Box charts are generated from technical replicates (n=16). (**e**) Numbers of integrated modulation parts in the first (R1), second (R2) and third (R3) round of screening (**Supplementary Data 3**).

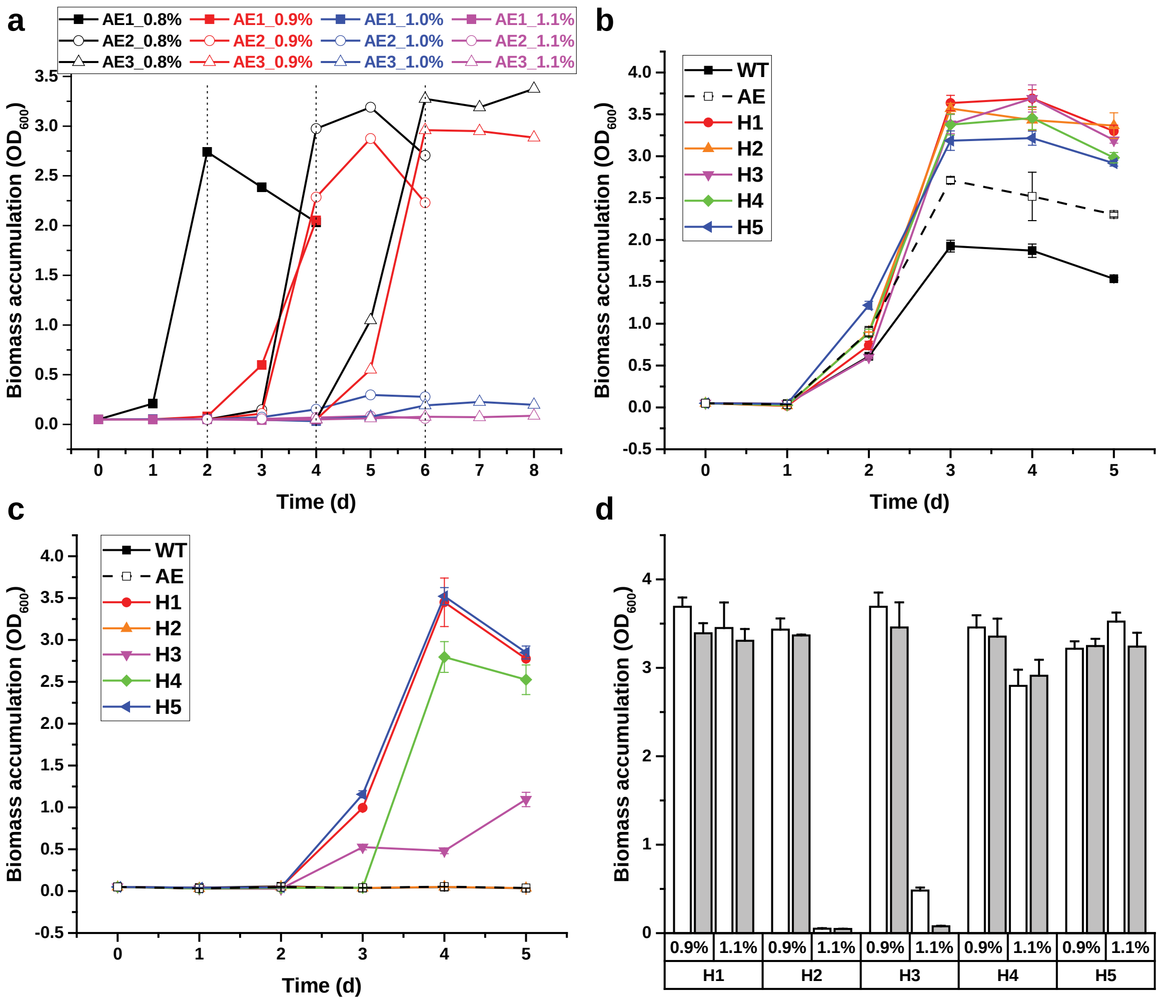

Supplementary Figure 7 | Cell growth of engineered yeast strains in HAc media. (**a**) Three rounds of adaptive evolution for the CAD-Cas9 parent strain were performed in SC media (pH=4) with 0.8% (black), 0.9% (Red), 1.0% (blue) and 1.1% (v/v) (magenta) HAc. Selection of cell cultures from the first (AE1, black square, 0.8%), second (AE2, red circle, 0.9%) and third (AE3, red triangle, 0.9%) rounds was indicated with vertical dashed lines. (**b**) Comparison of biomass accumulation of the parent strain (WT), adaptive population (AE) and individual mutant clones (H#) in the presence of 0.9% (v/v) HAc. (**c**) Comparison of biomass accumulation of the parent strain, adaptive population and individual mutant clones in the presence of 1.1% (v/v) HAc. (**d**) Strain stability test. For the individual clones (H1-H5) isolated after the third round of automated engineering, biomass accumulation in 0.9% and 1.1% (v/v) HAc media after 4 days was examined before (white) and after (grey) 100 generations of cell division in non-selective SC media. The same cultivation condition was used in (**d**) as in (**b**) and (**c**). Error bars represent the mean ± s.d. of technical replicates (n=3) and biological replicates (n=3) for adaptive population and all other strains, respectively.

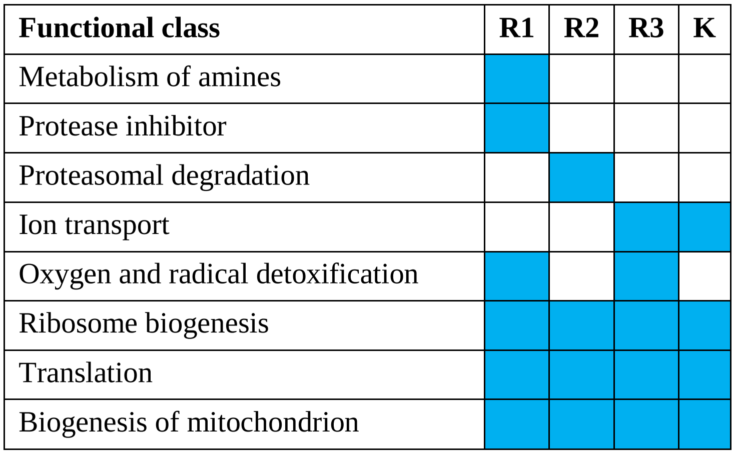

Supplementary Figure 8 | Functional clustering and statistics of integrated cassettes from different rounds of HAc tolerance screening. Genes enriched in each round were listed in **Supplementary Data 3**. Gene ontology enrichment analysis was performed according to the MIPS functional catalogue. R1, R2, R3 and K denote the first, second, third rounds of HAc engineering and known HAc resistance-relevant functional classes[1].

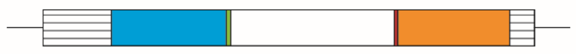

TEF1p-15bp-1-cDNA-15bp-2-PGK1t

PCR library construction

Extra bp | Tail (M13 F) | Barcode | PBS (TEF1p) | 15 bp-1 | 15 bp-2 | PBS (PGK1t)

PRO index primer F 5′ AGTAACGCCAGGTTTTCCCAGTCACGAC GCATTCGGCGTTGCATAGCAATCTAATCTAAG

......AGAAA GCATAGCAATCTAATCTAAG TTTTAATTACAA AAGCAGTGGTATCAA ......cDNA...... ......cDNA......TCTCATCGTACCCCGATTGAATTGAATTGAAATCGATAGATCAA......

......TCTTT CGTATCGTTAGATTAGATTC AAAATTAATGTT TTCGTCACCATAGTT ......cDNA...... ......cDNA......AGAGTAGCATGGGGCTAACTTAACTTAACTTTAGCTATCTAGTT......

CTTAACTTAACTTTAGCTATC 5′

PRO universal primer R

PCR Prpduct

AGTAACGCCAGGTTTTCCCAGTCACGAC GCATTCGGCGTTGCATAGCAATCTAATCTAAG TTTTAATTACAA AAGCAGTGGTATCAA ......cDNA...... ......cDNA......TCTCATCGTACCCCGATTGAATTGAATTGAAATCGATAG

TCATTGCGGTCCAAAAGGGTCAGTGCTG CGTAAGCCGCAA CGTATCGTTAGATTAGATTC AAAATTAATGTT TTCGTCACCATAGTT ......cDNA...... ......cDNA......AGAGTAGCATGGGGCTAACTTAACTTAACTTTAGCTATC

Normal TruSeq library construction

PCR Primer 1.0 (TruSeq libraries)

5′ AATGATACGGCGACCACCGA GATCTACACTCTTTCCCTACACGA

GATCTACACTCTTTCCCTACACGACGCTCTTCCGATCTAGTAACGCCAGGTTTTCCCAGTCACGAC GCATTCGGCGTTGCATAGCAATCTAATCTAAGTTTTAATTACAA AAGCAGTGGTATCAA ......cDNA...... AGATCGGAAGAGCACACGTCTGAACTCCAGTCAC_NNNNNN_ ATCTCGTATGCCGTCTTCTGCTTG

CTAGATGTGAGAAAGGGATGTGCTGCGAGAAGGCTAGA TCATTGCGGTCCAAAAGGGTCAGTGCTG CGTAAGCCGCAA CGTATCGTTAGATTAGATTC AAAATTAATGTT TTCGTCACCATAGTT ......cDNA...... TCTAGCCTTCTCGTGTGCAGACTTGAGGTCAGTG_NNNNNN_ TAGAGCATACGGCAGAAGACGAAC

TAGAGCATACGGCAGAAGACGAAC 5′

PCR Primer 2.0 (TruSeq libraries)

PCR Primer 1.0 (TruSeq libraries)

AATGATACGGCGACCACCGA GATCTACACTCTTTCCCTACACGA

GATCTACACTCTTTCCCTACACGACGCTCTTCCGATCT ......cDNA...... AGATCGGAAGAGCACACGTCTGAACTCCAGTCAC_NNNNNN_ ATCTCGTATGCCGTCTTCTGCTTG

CTAGATGTGAGAAAGGGATGTGCTGCGAGAAGGCTAGA ......cDNA...... TCTAGCCTTCTCGTGTGCAGACTTGAGGTCAGTG_NNNNNN_ TAGAGCATACGGCAGAAGACGAAC

TAGAGCATACGGCAGAAGACGAAC

PCR Primer 2.0 (TruSeq libraries)

Custom TruSeq library construction

PCR Primer 1.0 custom1 (TruSeq libraries)

5′ AATGATACGGCGACCACCGA GATCTACACTCTTTCCCTACACGACGCTCTTCCGATCT CCAGGTTTTCCCAGTCACGAC

AGTAA CGCCAGGTTTTCCCAGTCACGAC GCATTCGGCGTTGCATAGCAATCTAATCTAAGTTTTAATTACAA AAGCAGTGGTATCAA ......cDNA...... AGATCGGAAGAGCACACGTCTGAACTCCAGTCAC_NNNNNN_ ATCTCGTATGCCGTCTTCTGCTTG

TCATT GCGGTCCAAAAGGGTCAGTGCTG CGTAAGCCGCAA CGTATCGTTAGATTAGATTC AAAATTAATGTT TTCGTCACCATAGTT ......cDNA...... TCTAGCCTTCTCGTGTGCAGACTTGAGGTCAGTG_NNNNNN_ TAGAGCATACGGCAGAAGACGAAC

TAGAGCATACGGCAGAAGACGAAC 5′

PCR Primer 2.0 (TruSeq libraries)

Sequencing Primer (TruSeq libraries)

5' ACACTCTTTCCCTACACGACGCTCTTCCGATCT

Index Read Primer (TruSeq libraries)

GATCGGAAGAGCACACGTCTGAACTCCAGTCAC

AATGATACGGCGACCACCGA GATCTACACTCTTTCCCTACACGACGCTCTTCCGATCT CCAGGTTTTCCCAGTCACGAC GCATTCGGCGTTGCATAGCAATCTAATCTAAGTTTTAATTACAA AAGCAGTGGTATCAA ......cDNA...... AGATCGGAAGAGCACACGTCTGAACTCCAGTCAC_NNNNNN_ ATCTCGTATGCCGTCTTCTGCTTG

TTACTATGCCGCTGGTGGCT CTAGATGTGAGAAAGGGATGTGCTGCGAGAAGGCTAGA CGTCCAAAAGGGTCAGTGCTG CGTAAGCCGCAA CGTATCGTTAGATTAGATTC AAAATTAATGTT TTCGTCACCATAGTT ......cDNA...... TCTAGCCTTCTCGTGTGCAGACTTGAGGTCAGTG_NNNNNN_ TAGAGCATACGGCAGAAGACGAAC

P5

P7

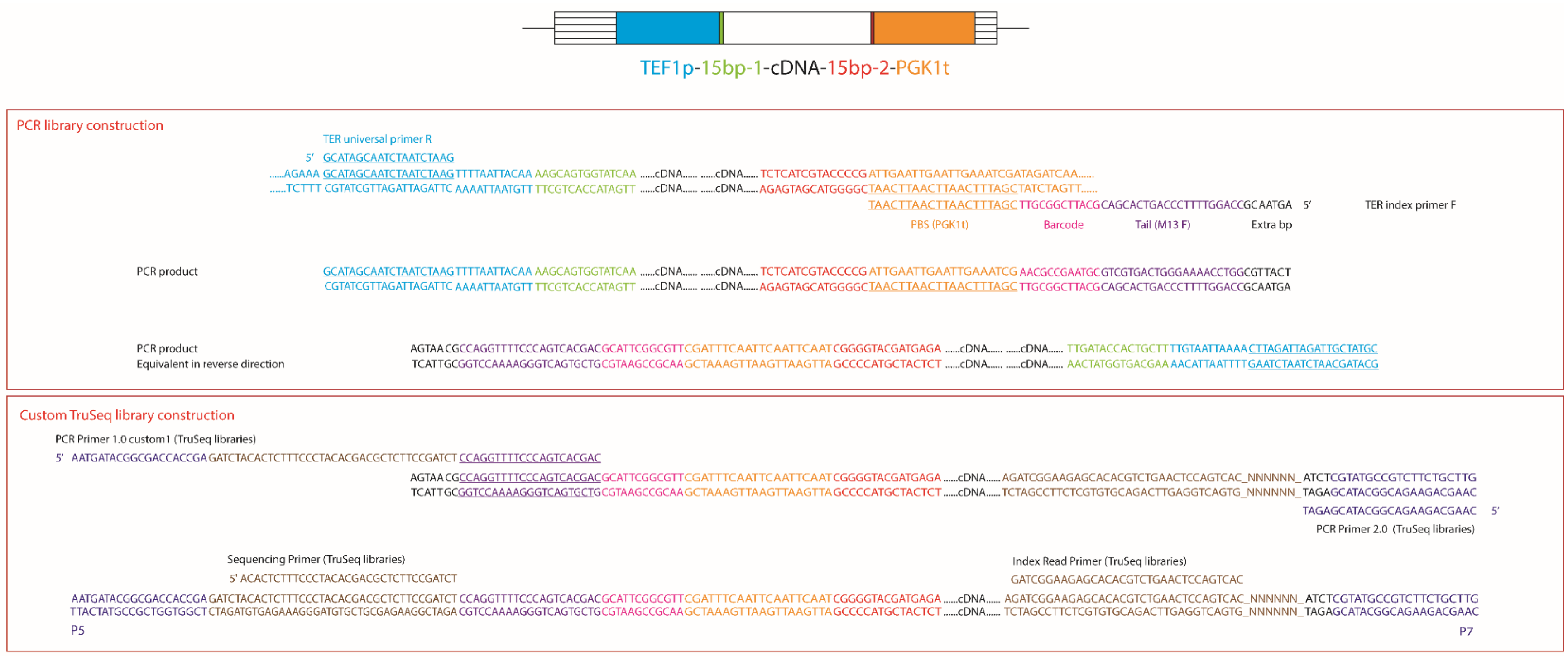

Supplementary Figure 9 | Scheme of modified TruSeq workflow for NGS analysis of PCR libraries. The designs to enrich junction fragments near the promoter and terminator are explained in (**a**) and (**b**), respectively. The color code—cyan: promoter; orange: terminator; green: 15 bp handle#1; red: 15 bp handle#2; Indigo: custom sequence M13F; purple: 12 bp barcode; brown: Truseq adaptors; blue: sequence to bind flow cells. PCR library construction shows the process to PCR-amplify modulation parts either from plasmid or genomic DNA samples. Normal TruSeq library construction shows the results when the original PCR primers "PCR Primer 1.0" and "PCR Primer 2.0" were used to construct the shortgun DNA libraries. Custom TruSeq library construction shows enrichment of junction fragments and inclusion of compatible sequences for the Truseq protocol via the use of a custom primer "PCR Primer 1.0 custom1". "Sequencing Primer" and "Index Read Primer" are original sequences from the Truseq kit.

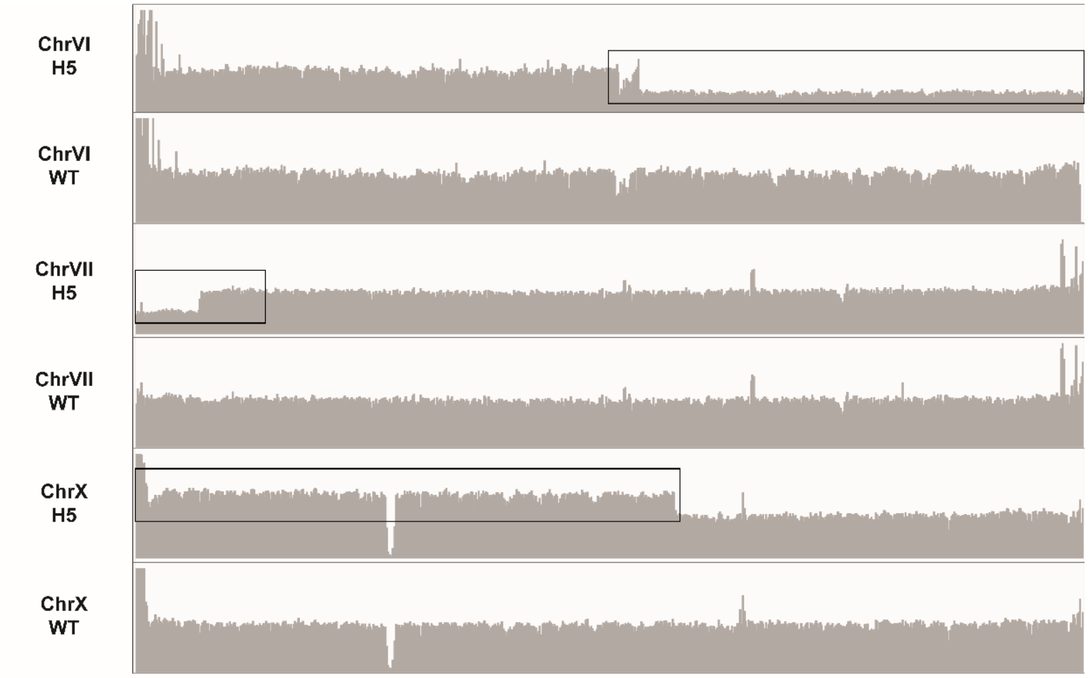

Supplementary Figure 10 | Large-scale genomic rearrangement in the H5 mutant visualized by read coverage in Integrative Genomics Viewer[2].

Supplementary Table 1 | Insets of 20 random plasmids from the modulation part library

Overexpression library		Knockdown library	
Gene name	ORF length (bp)	Gene name	ORF length (bp)
CBK1	2271	*RPS4A*	786
OLE1	1533*	*TOS6*	309
RPS8B	604	*INH1*	258
HSC82	2118	*KAR2*	2049
SCD6	1050	*GPM1*	744
ATP17	306	*SPI1*	447
UFD1	1086	*SSA4*	1929
HSP26	645	*RIB4*	510
LSP1	1026	*GIM3*	390
HTB1	396	*PIL1*	1020

* A fragment (1-264bp) of the *OLE1* ORF was cloned. The fragment had the characteristic poly-A tail in an mRNA molecule, indicating a premature transcriptional stop instead of inability of the cDNA synthesis kit to amplify the 5’ end of the mRNA for cloning a full-length cDNA.

Supplementary Table 2 | Serial transfer to enrich yeast mutants with enhance NVA resistance and glycerol utilization.

	Norvaline resistance			Glycerol utilization			
Round	Concentration (g/L)	Time to reach OD_{600}=2.5 (day)		Inoculum (%)		Time to reach OD_{600}=1(day)	
		Library	Control	Library	Control	Library	Control
1	4	1.5	2.5	1	1	2.5	3.5
2	4	1	1.5	1	1	2	3
3	4	1	1.5	1	1	2	2.5
4	5	1	1.5	0.1	1	2.5	2.5
5	5	1	1.5	0.1		1.5	
6	6	1	1.5				
7	6	1	1				

Supplementary Table 3 | Known gene functions targeted by isolated modulation parts.

Gene	Description (www.yeastgenome.com)
get3	Guanine nucleotide exchange factor for Gpa1p; amplifies G protein signaling; functions as a chaperone under ATP-depleted oxidative stress conditions; subunit of GET complex, involved in ATP dependent Golgi to ER trafficking and insertion of tail-anchored (TA) proteins into ER membrane under non-stress conditions; binds as dimer to transmembrane domain (TMD) cargo, shielding TMDs from aqueous solvent; protein abundance increases under DNA replication stress
sdh3	Subunit of succinate dehydrogenase and of TIM22 translocase; functions as cytochrome b subunit of succinate dehydrogenase, which couples oxidation of succinate to transfer of electrons to ubiquinone as part of the TCA cycle and the mitochondrial respiratory chain; also required for mitochondrial inner membrane protein import as part of the TIM22 complex
pex5	Peroxisomal membrane signal receptor for peroxisomal matrix proteins; receptor for the C-terminal tripeptide signal sequence (PTS1) of peroxisomal matrix proteins; required for peroxisomal matrix protein import; also proposed to have PTS1-receptor independent functions
ent3	Protein containing an N-terminal epsin-like domain; involved in clathrin recruitment and traffic between the Golgi and endosomes; associates with the clathrin adaptor Gga2p
pti1	Essential component of CPF (cleavage and polyadenylation factor); involved in 3' end formation of snoRNA and mRNA; interacts directly with Pta1p; relocalizes to the cytosol in response to hypoxia; similar to mammalian Cleavage-Stimulation Factor CstF-64
idh2	Subunit of mitochondrial NAD(+)-dependent isocitrate dehydrogenase; complex catalyzes the oxidation of isocitrate to alpha-ketoglutarate in the TCA cycle; phosphorylated
tar1	Protein potentially involved in regulation of respiratory metabolism; located in the mitochondria; interacts genetically with RPO41 and physically with Coq5p; encoded within the 25S rRNA gene on the opposite strand
sed1[#]	Major stress-induced structural GPI-cell wall glycoprotein; associates with translating ribosomes, possible role in mitochondrial genome maintenance; ORF contains two distinct variable minisatellites
ych1	Phosphatase with sequence similarity to Cdc25p; Arr2p and Mih1p; member of the single-domain rhodanese homology superfamily
sol3	6-phosphogluconolactonase; catalyzes the second step of the pentose phosphate pathway; weak multicopy suppressor of los1-1 mutation
bul1[$]	Ubiquitin-binding component of the Rsp5p E3-ubiquitin ligase complex; disruption causes temperature-sensitive growth; overexpression causes missorting of amino acid permeases
cit1[*]	Citrate synthase; catalyzes the condensation of acetyl coenzyme A and oxaloacetate to form citrate; the rate-limiting enzyme of the TCA cycle; nuclear encoded mitochondrial protein
afg1	Protein that may act as a chaperone for cytochrome c oxidase subunits; conserved protein; may act as a chaperone in the degradation of misfolded or unassembled cytochrome c oxidase subunits; localized to matrix face of the mitochondrial inner membrane; member of the AAA family but lacks a protease domain

[#]The *SED1* gene encodes a stress-induced structural cell wall protein Sed1p, which is anchored on the cell surface through a glycosylphosphatidylinositol (GPI) domain[3]. It has been shown that the use of the Sed1p anchoring domain enabled efficient surface display of heterologous proteins in yeast[4], suggesting high displaying levels of Sed1p. Hence, it is possible that suppressed Sed1p expression can improve the capacity of cell surface to accommodate other cell wall proteins, including the EGII-agglutinin fusion.

[*]The knockdown cassette of *CIT1* targeted the tricarboxylic acid (TCA) pathway, and reduced TCA activities has been linked to fast glycerol metabolism in *E. coli*[5].

[$]Knockdown of the *BUL1* gene may lead to mistargeting of the general amino acid permease Gap1p, and reduced expression of the Gap1p was found to increase isobutanol titer[6]

Supplementary Table 4 | Multiplex integration cassettes from yeast mutants engineered using the biological foundry

G6		G1*			H5	
		YAL033W	YKR070W	YLR262C-A	YAL033W	YKR070W
		YBR009C	YKR095W	YML028W	YBR009C	YKR095W
YBR082C	YLR204W	YBR010W	YLL009C	YML030W	YBR010W	YLL009C
YDL125C	YML028W	YDL136W	YLR287C-A	YMR117C	YDL136W	YLR287C-A
YDR063W	YMR119W	YDL184C	YML028W	YMR193W	YDL184C	YML028W
YDR296W	YMR193W	YDL191W	YMR117C	YNL055C	YDL191W	YMR117C
YEL052W	YOL016C	YDR050C	YMR193W	YNR001C	YDR050C	YMR193W
YER158W-A	YOL143C	YDR363W-A	YNR001C	YOL016C	YDR363W-A	YNR001C
YHR005C	YOR020C	YEL052W	YOR020C	YOR020C	YEL052W	YOR020C
YJL144W	YOR293W	YER030W	YPL225W	YPL037C	YER030W	YPL225W
		YGR076C	YPR010C-A	YPR010C-A	YGR076C	YPR010C-A
		YGR243W		YPR151C	YGR243W	

Overexpression cassettes were underlined. Blue font indicates both overexpression and knockdown parts were identified, a phenomenon that was also observed in a previous report isolating both up- and down-regulation of the same gene in a genome-wide screening in *E. coli*[7]. All others are knockdown cassettes.

Supplementary Table 5 | DNA sequences included in this study

**Primer sequences for cloning**	
**Name**	**DNA sequence (5'→3')**
PR361	ATGACAAGGGTGACGCATTGG
PR362	CTAAACCCACACCGGGTG
PR364	ATCACGAGGCCCTTTTCTTTGAAAAGATAATGTATG
PR365	CAGACAAGCTGTGACAGACATAAAAAACAAAAAAAG
PR346	AAACGAAGTTCTCCTCGAGGATATG
PR347	AAAACATATCCTCGAGGAGAACTTC
PR432	AATACCGCACAGATGCGTAAGGAGAAAATACCGCATCAGG**GAAGTTCTCCTCGAGGATATAGG**TGTTGGAATAGAAATCAACTATCATCTACTAACTAGTATTTACATTACTAGTATATTATCATATACGGTGTTAGAAGATGACGCAAATGATGAGAAATAGTCATCTAAATTAGTGGAAGCTGAAACGCAAGGATTGATAATGTAATAGGATCAATGAATATAAACATATAAAATGATGATAATAATATTTATAGAATTGTGTAGAATTGCAGATTCCCTTTTATGGATTCCTAAATCCTTGAGGA*GTTTAAAC*GAACTTCTAGTATATTCTGTATACCTAATATTATAGCCTTTATCAACAATGGAATCCCAACAATTATCTCAACATTCACCCATTTCTCA**GAAGTTCTCCTCGAGGATATAGG**TCACTGCCCGCTTTCCAGTCGGGAAACCTGTCGTGCCAGC
PR436	CCTGATGCGGTATTTTCTCC
PR437	CCAGTCGGGAAACCTGTCG
PR438	CGACAGGTTTCCCGACTGG
PR451	GATTCCTAAATCCTTGAGGAACACACCATAGCTTCAAAATG
PR452	ACAGAATATACTAGAAGTTCCAGGAAGAATACACTATAC
PR511	CGGGAAACCTGTCGTGCCAGTAGGTCTAGAGATCTGTTTAGC
PR512	GTTATGTTCAATTGGCAGATCATTAAGGGTTCTCGAGAGC
PR513	GCTCTCGAGAACCCTTAATGATCTGCCAATTGAACATAAC
PR514	ATTCATTAATGCAGCCGCGGTCTTTCTGTATCGCAAATAAG
PR515	CGGGAAACCTGTCGTGCCAGACATCCGAACATAAACAACC
PR516	CCGCGGCTGCATTAATG
PR608	AGTTTTAATTACAAA **AAGCAGTGGTATCAA** *GTTTAAAC* CAGGAAGGGATGGCTGAGG
PR609	TCAATTCAATTCAAT **CGGGGTACGATGAGA** *GTTTAAAC* GAGCTCTAGAGATATCGTCG
PR610	AGTTTTAATTACAAA **CGGGGTACGATGAGA** *GTTTAAAC* CAGGAAGGGATGGCTGAGG
PR611	TCAATTCAATTCAAT **AAGCAGTGGTATCAA** *GTTTAAAC* GAGCTCTAGAGATATCGTCG
PR615	AATCTAAGTTTTAATTACAAAAAGCAGTGG
PR616	TCGATTTCAATTCAATTCAATCGGGGTACG
PR617	AATCTAAGTTTTAATTACAAACGGGGTACG
PR618	TCGATTTCAATTCAATTCAATAAGCAGTGG
PR1001	AATCTAAGTTTTAATTACAAAATGAAGGTTTTGATTGTCTTGTTGGC
PR1002	TTGGCGCTAGAACCTCCACCGCCACT
PR1003	GAGGTTCTAGCGCCAAAAGCTCTTTTATC
PR1004	CGATTTCAATTCAATTCAATTTAGAATAGCAGGTACGACAAAAG
Primers for qPCR	
ACT1 Rev	TCAAAGAAGCCAAGATAGAACCA
qPCR ACT1 For	AATTCGTTGTAGAAGGTATG
qPCR ACT1 Rev	ATACCTGGGAACATGGTGGT

SED1 Rev	GAAGAAGCAGAGGATGAAACT
qPCR SED1 For	CCAACAGTACATCTGCTTCTTCCA
qPCR SED1 Rev	TGTCGGATTCTGGAGCTTCAG
YCH1 Rev	AACGCCACAGATCGGGTAGG
qPCR YCH1 For	ATACCTGGATCCGACTGAATTG
qPCR YCH1 Rev	TGCCATCCGTCCTTGATATG
SOL3 Rev	CCAAAAGCTTCCTCATTAACG
qPCR SOL3 For	TTTCTGAGAGGGCTAGTTTGAC
qPCR SOL3 Rev	TCAAAGAGCCACCGCTAAC
BUL1 Rev	TACTCGAACGCAATAAAACC
qPCR BUL1 For	TAGGCAGAAACAAGAGGGTG
qPCR BUL1 Rev	ATGAGAATGTGCTAGTTCGGG
CIT1 Rev	CGTAATCTGGGAAATGTTTC
qPCR CIT1 For	TTTGGTTGCTTTTGACTGGTG
qPCR CIT1 Rev	TGGGAGGCTATCTAAAAGTTGG
AFG1 Rev	CTTCATCAAGAGCAAACATC
qPCR AFG1 For	GCCGCAGAGATTGCAAATAAT
qPCR AFG1 Rev	CGGATAGTAAGGCAGTCATCAG
qPCR ALG9 For	CACGGATAGTGGCTTTGGTGAACAATTAC
qPCR ALG9 Rev	TATGATTATCTGGCAGCAGGAAAGAACTTGGG
qPCR GFP For	GATGGTGATGTTAATGGGCAC
qPCR GFP Rev	GGGTAAGTTTTCCGTATGTTGC

**Primers for NGS analysis**			
Barcode name	Sample name	PRO index primer F (5'→3')	TER index primer F (5'→3')
Color code for forward primers		**extra bp-M13F-12bp barcode-Primer binding site (promoter)**	**extra bp-M13F*-12bp barcode-Primer binding site (terminator)**
806rcbc1631	Integrated library	AGTCACGCCAGGTTTTCCCAGTCACGACGGCAAGGCACAAGCATAGCAATCTAATCTAAG	AGTCACGCCAGGTTTTCCCAGTCACGACGGCAAGGCACAACGATTTCAATTCAATTCAAT
806rcbc1001	R1	AGTCACGCCAGGTTTTCCCAGTCACGACGGACCGCTTTCAGCATAGCAATCTAATCTAAG	AGTCACGCCAGGTTTTCCCAGTCACGACGGACCGCTTTCACGATTTCAATTCAATTCAAT
806rcbc609	R2	AGTCACGCCAGGTTTTCCCAGTCACGACGCAAGCTGTCTCGCATAGCAATCTAATCTAAG	AGTCACGCCAGGTTTTCCCAGTCACGACGCAAGCTGTCTCCGATTTCAATTCAATTCAAT
806rcbc441	R3	AGTCACGCCAGGTTTTCCCAGTCACGACCATACACGCACCGCATAGCAATCTAATCTAAG	AGTCACGCCAGGTTTTCCCAGTCACGACCATACACGCACCCGATTTCAATTCAATTCAAT
806rcbc1333	H5	AGTCACGCCAGGTTTTCCCAGTCACGACGCATCTAAAGCCGCATAGCAATCTAATCTAAG	AGTCACGCCAGGTTTTCCCAGTCACGACGCATCTAAAGCCCGATTTCAATTCAATTCAAT
806rcbc930	Plasmid library	AGTCACGCCAGGTTTTCCCAGTCACGACGCACTTCATTTCGCATAGCAATCTAATCTAAG	AGTCACGCCAGGTTTTCCCAGTCACGACGCACTTCATTTCCGATTTCAATTCAATTCAAT
806rbcb756	Glycerol Enriched plasmid	AGTCACGCCAGGTTTTCCCAGTCACGACACCGTCTTTCTCGCATAGCAATCTAATCTAAG	AGTCACGCCAGGTTTTCCCAGTCACGACACCGTCTTTCTCCGATTTCAATTCAATTCAAT
Reverse primer		PRO universal primer R (5'→3')	TER universal primer R (5'→3')
		CGATTTCAATTCAATTCAAT	GCATAGCAATCTAATCTAAG

Sequence features to determine modulation modes			
Modulation mode	Combination	DNA sequences of the combinations	Following sequence relative to an ORF
Overexpression	P_{TEF1}-15bp-1	TTTTAATTACAAAAAGCAGTGGTATCAA	Upstream or the start
	T_{PGK1}-15bp-2	TCAATTCAATTCAATCGGGGTACGATGAGA	Downstream or the end
Knockdown	P_{TEF1}-15bp-2	TTTTAATTACAAACGGGGTACGATGAG	Downstream or the end
	T_{PGK1}-15bp-1	TCAATTCAATTCAATAAGCAGTGGTATCAA	Upstream or the start

Supplementary References

1. Mira, N.P., Palma, M., Guerreiro, J.F. & Sa-Correia, I. *Microb Cell Fact* **9**, 79 (2010).
2. Robinson, J.T. et al. *Nat Biotechnol* **29**, 24-26 (2011).
3. Shimoi, H., Kitagaki, H., Ohmori, H., Iimura, Y. & Ito, K. *J Bacteriol* **180**, 3381-3387 (1998).
4. Inokuma, K., Hasunuma, T. & Kondo, A. *Biotechnol Biofuels* **7**, 8 (2014).
5. Martinez-Gomez, K. et al. *Microb Cell Fact* **11**, 46 (2012).
6. Dundon, C.A., Smith, C., Nahreini, P., Thevelein, J. & Saerens, S. *United States patent* US 13701247 A2 (2013).
7. Warner, J.R., Reeder, P.J., Karimpour-Fard, A., Woodruff, L.B. & Gill, R.T. *Nat Biotechnol* **28**, 856-862 (2010).
